# Tell me the match context, and I’ll tell you how much the team will run, or should have run: an update and divisional extension of a predictive running model for Spanish professional football

**DOI:** 10.5114/biolsport.2026.164806

**Published:** 2026-09-11

**Authors:** Julen Castellano, Roberto López-Del Campo, Ricardo Resta, Raul Hileno

**Affiliations:** 1 University of the Basque Country, (UPV/EHU), Vitoria-Gasteiz, Spain; 2 Department of Competitions and Mediacoach, LaLiga, Madrid, Spain; 3 INEFC Lleida. University of Lleida, Spain

**Keywords:** Match running performance, Predictive modelling, Regression analysis, Situational variables, Competition level, Soccer

## Abstract

To update the predictive model of a team’s total distance (TotDisTea) proposed by Castellano et al. (2024) for the Spanish First Division (LaLiga1), using five seasons (2021–22 to 2025–26), and to extend it to the Second Division (LaLiga2), from a dual perspective: a retrospective (evaluative) approach to assess performance already produced, and a prospective (predictive) approach usable before a match. Team physical performances from official matches were analysed (LaLiga1: 2,894 team-match records from 1,447 matches; LaLiga2: 3,542 from 1,771), recorded with a multi-camera system. For each division, an all-possible-regressions procedure selected the best linear model for TotDisTea from seven situational predictors: match location, match outcome, team and opponent level, effective-playing-time (EffPlaTim), ball-possession (BalPos) and the opponent’s total distance (TotDisOpp). A reduced model excluding TotDisOpp was additionally estimated for prospective use. Models were cross-validated at the match level and fitted with cluster-robust standard errors. In both divisions TotDisOpp was the strongest predictor (b= 0.72 and 0.79). The LaLiga1 model additionally retained team and opponent levels, EffPlaTim and BalPos (R^2^adj= 0.86); the more parsimonious LaLiga2 model retained TotDisOpp, EffPlaTim and BalPos (R^2^adj= 0.85). Match location and match outcome were not selected in either league. The reduced models retained EffPlaTim and BalPos, with lower accuracy (R^2^adj= 0.71 and 0.61). Opponent running is the most stable predictor in both divisions, but the predictive structure is league-specific and coefficients may change over time, warranting periodic updating. The equations let practitioners evaluate a team’s response after a match, and enable anticipation beforehand.

## INTRODUCTION

Elite football is not a static phenomenon: its physical demands have evolved steadily over recent decades, to a greater or lesser extent, across every league that has been examined [1]. In the English Premier League, high-intensity distance and actions increased by roughly 30% over seven seasons while total distance decreased by around 2% [2], a trend that was confirmed when the data were disaggregated by playing position [3] and that has remained in force in more recent seasons [4–6]. A comparable, and rapidly expanding, body of evidence has emerged in Spain with a proliferation of studies on match physical performance, including differences in running and metabolic-load demands between competitive levels [7, 8]; the characterisation of playing positions and acceleration–speed profiles [9]; the influence of contextual and strategic variables on the physical response [10]; the impact of rule and competition changes such as the Video Assistant Referee [11] and of mid-season coaching changes [12]; the relationship between offensive team variables and goal scoring [13] and the demands faced by players promoted from the Second to the First Division [14].

Importantly, the continuity and granularity of these databases have made longitudinal designs feasible, and it is precisely these multi-season analyses that are revealing how the game is changing. Over an eight-season window, the technical–tactical and physical profile of LaLiga teams showed measurable shifts, with a tendency to prioritize defensive organisation [15], and changes in the physical and technical profile have likewise been documented when players step up between divisions [14]. Comprehensive ten-year [13] and multivariate single-season approaches [16] have refined our understanding of which offensive and external-load variables matter most, while four-season comparisons of the top two leagues [17, 18] have shown not only that physical performance is higher in the First Division but that it has increased over time in both categories, especially in the most recent seasons, in total volume as well as in high-intensity distances and the number of sprints.

Within this scenario, the predictive model proposed by Castellano et al. [19] to estimate the total distance covered by a team, supported by contextual variables such as the distance run by the opponent, effective playing time, and ball-possession, was built with data spanning the 2016–17 to 2019–20 seasons. Given that match demands have continued to change since then, its coefficients require updating if the model is to remain useful for anticipating and evaluating physical performance in competition. Indeed, before developing a new model, we first applied the original coefficients to the current dataset as an external test. This exercise revealed a systematic bias: in practically all matches the model predicted a lower total distance than the one actually recorded, a discrepancy that drew the authors’ attention and provided direct empirical evidence that the previous coefficients no longer reflect present-day demands, rather than merely assuming so from contextual change. Moreover, because the First and Second Divisions differ systematically in physical demands and, above all, in how these demands have evolved over time, merging both competitions into a single model risks masking these particularities and biasing predictions for either category.

For all of the above, the purpose of this study was twofold. First, to update the predictive model of total distance covered by a team in the Spanish First Division, originally proposed by Castellano et al. [19], using data from the last five seasons (2021–22 to 2025–26); and, second to extend this approach to the Second Division by building an analogous, category-specific model. We hypothesised that the updated models would differ from the original model and from each other across divisions, primarily in the set of predictors retained and, to a lesser extent, in the magnitude of their coefficients. Given that a formal inferential comparison against the previously published model was not feasible, and that coefficient magnitudes were examined under cluster-robust inference, these differences were assessed descriptively rather than through a formal inferential test.

## MATERIALS AND METHODS

### Approach to the problem

A retrospective observational study based on aggregated data from the match-tracking records of official matches in the top two Spanish Professional Men’s Football divisions (LaLiga1, the First Division and LaLiga2, the Second Division) over five seasons (from 2021–22 to 2025–26), which are managed by the company *Mediacoach*^®^, currently integrated within LaLiga’s Performance environment.

### Participants

Physical performance data were recorded for each team in each match and were separated by league and season. To train the model, all matches played in LaLiga1 (n = 3,800 team–match records; 10 matches × 2 teams × 38 matchdays × 5 seasons) and in LaLiga2 (n = 4,620 team–match records;

11 matches × 2 teams × 42 matchdays × 5 seasons) were used. LaLiga2 promotion play-off matches were excluded. After removing records containing data errors (< 1%) and matches involving a sending-off, the coverage achieved in each division was ≈76% (2,894 and 3,542 team-match records for LaLiga1 and LaLiga2, respectively). These exclusions followed predefined criteria applied uniformly across both divisions, ensuring a consistent and comparable sample. In accordance with its ethical guidelines, this investigation does not include information that identifies football players. Data were treated in accordance with the Declaration of Helsinki and the study was approved by the Ethics Committee on Research on Human Beings (CEISH) of the University (code: M10-2024-124).

### Variables

The seven predictors from the previous work by Castellano and colleagues [19] were retained: match location (MatLoc), match outcome (MatOut), team level (TeaLev), opponent level (OppLev), effective playing time (EffPlaTim), ball-possession time (BalPos), and distance covered by the opponent during effective playing time (TotDisOpp, in km). For the team levels in LaLiga1, the classification proposed in the previous study was kept: level 1, Relegation (positions 18^th^– 20^th^); level 2, Remained (positions 8^th^–17^th^); level 3, Europa League (positions 5^th^–7^th^); and level 4, Champions League (positions 1^st^–4^th^). In the Second Division, where there are no European qualification places and the league comprises 22 teams, the team and opponent levels (TeaLev and OppLev) were operationalised into four categories according to the final standings: level 1 (positions 19^th^–22^nd^, i.e. relegation), level 2 (positions 13^th^–18^th^), level 3 (positions 7^th^–12^th^), and level 4 (positions 1^st^–6^th^, i.e. direct promotion and play-off contenders). As in the First Division, the category with the lowest numerical value was used as the reference category in the regression. Because these categories are derived from each team’s final league position, competitive level constitutes a season-level contextual classification rather than a value necessarily available before each individual match, and is therefore suited to retrospective analysis.

### Procedures

Time-motion data were recorded using the TRACAB Gen5 multicamera tracking system (EA SPORTS). Ball-possession time was obtained through OPTA^®^ Sportsdata Company (Opta Sports, London, UK). Both TRACAB and OPTA are managed by the *Mediacoach*^®^ software. The reliability of the OPTA system has been previously demonstrated [20], as has that of the TRACAB video-tracking system for quantifying physical demand [21, 22], showing good-quality data. The same recording system and data-collection procedure were used in both divisions and throughout all the seasons analysed, ensuring homogeneity in data collection. The reports generated were exported to Microsoft Office Excel (Microsoft Corporation, Washington, USA) for further processing.

### Statistical analysis

Because the two professional divisions were analysed separately, the whole modelling process was carried out independently for the LaLiga1 and LaLiga2 divisions. For each division, the all-possible-regressions procedure was used to select the best predictive linear regression model for the team’s total distance covered (TotDisTea). No stepwise selection procedures were applied, given their well-known limitations [23]. This procedure constructs every possible sub-model by combining the predictors of the maximum model and assesses the degree to which each one meets the established selection criteria [24]. The maximum model included seven predictor blocks (Mat-Loc, MatOut, TeaLev, OppLev, EffPlaTim, BalPos and TotDisOpp), two of which (TeaLev and OppLev) were categorical variables entered as sets of dummy indicators. The selection criteria were: (a) the principle of parsimony; (b) a small Mallows’ Cp value; and (c) a high adjusted coefficient of determination (R^2^adj). Mallows’ Cp was calculated as SSres,p/σ^−2^ – (n – 2p), with σ^−2^ estimated from the maximum model (SSres,full/(n – p_full)) and p denoting the number of parameters including the intercept. Lower Cp values indicate a better trade-off between goodness of fit and model complexity. For each division, all candidate sub-models were ranked in ascending order of Cp, and the sub-model with the smallest Cp value was taken as the best-fitting model; among sub-models with comparably low Cp, the most parsimonious solution with the highest R^2^adj was preferred. In addition to this full model, and to enable a prospective (pre-match) application, a reduced model was estimated for each division following the identical procedure but excluding TotDisOpp from the candidate predictors, since the opponent’s in-match distance is not available before kick-off.

Once the best model had been selected in each division, the reliability of its predictions was assessed by cross-validation. Prior to model estimation, the outcome variable (and, where applicable, the opponent’s corresponding value) was screened for outlying observations using the interquartile-range criterion (values below Q1 − 1.5 × IQR were excluded), and the resulting cleaned dataset was used for all candidate models within that division. The selected models were then checked for the following assumptions (the statistics and plots used are specified in parentheses): (a) absence of collinearity (variance inflation factor); (b) normality of the residuals (histogram and Q–Q plot of the standardized residuals); and (c) linearity of the relationship and homogeneity of the variance of the residuals (scatter plot of the residuals against the predicted values). After checking the diagnostics, the regression coefficients (b) and their 95% confidence intervals were estimated, and the resulting equation was used to predict the outcome for selected value patterns of the predictor variables.

All statistical analyses were performed in Python (version 3.12; Python Software Foundation, Wilmington, DE, USA). The pandas (v2.0.3) and NumPy (v1.24.4) libraries were used for data handling and numerical computation. Multiple linear regression models were fitted by ordinary least squares using NumPy’s linear-algebra routines (np.linalg.lstsq). Because each match contributes two paired team-match observations and teams provide repeated measures across matches and seasons, standard errors and 95% confidence intervals were computed using cluster-robust (sandwich) estimators with two-way clustering by match and by team, rather than from the classical OLS covariance; the corresponding t-tests and confidence intervals were based on these robust standard errors, using the t-distribution as implemented in the SciPy library (v1.10.1). Variance inflation factors were derived analytically from the OLS solution. The all-possible-regressions procedure was implemented with custom code (itertools), enumerating every combination of predictor blocks, and candidate models were compared using Mallows’ Cp and adjusted R^2^. Model stability was assessed through 10-fold cross-validation implemented at the match level (GroupKFold, scikit-learn v1.3.2), so that both team-match observations from a given match were always allocated to the same fold and no match was split between training and test sets. This cross-validation procedure was applied separately to the full and reduced models within each division. In each fold, the corresponding model was re-estimated on the training partition and evaluated on the held-out partition, and the cross-validated R^2^ was computed as the mean across folds. The significance level was set at p ≤ .05.

## RESULTS

### Retrospective models including opponent’s distance

Across the analysed matches, TotDisTea averaged 85.1 km (SD = 6.4) in LaLiga1 and 83.5 km (SD = 5.7) in LaLiga2 (ranges 67.4–107.1 and 67.6–101.3, respectively). Relative to this variability, the models’ prediction error was small: the RMSE represented 2.8% of the mean in LaLiga1 and 2.6% in LaLiga2 (≈ 0.37–0.38 SD of the outcome).

A separate predictive model for the team’s total distance covered (TotDisTea) was estimated for each division. Both models explained a large proportion of the variance in TotDisTea and cross-validated well (LaLiga1: R^2^adj = 0.86, cross-validated R^2^ = 0.86; LaLiga2: R^2^adj = 0.85, cross-validated R^2^ = 0.85), but they differed in which predictors were retained: TotDisOpp was the strongest predictor in both divisions, whereas TeaLev and OppLev were retained only in LaLiga1, resulting in a more parsimonious model for LaLiga2. The coefficients of both models, together with their robust standard errors, 95% confidence intervals, t-statistics and p-values, are reported in Table 1.

**TABLE 1:** Regression coefficients (b), robust standard errors (SE), 95% confidence intervals (CI), t-statistics and p-values for the full (retrospective) and reduced (prospective) models in each division. Standard errors and confidence intervals are cluster-robust (two-way clustering by match and team).

Model	Division	Predictor	b	SE	95% CI	t	p
retrospective	LaLiga1	Constant	7.797	1.40	[4.913, 10.681]	5.57	< .001
		TeaLev2	0	0.36	[-0.748, 0.748]	0	1
		TeaLev3	-0.186	0.58	[-1.371, 1.000]	-0.32	0.75
		TeaLev4	-0.427	0.98	[-2.454, 1.599]	-0.43	0.67
		OppLev2	-0.020	0.08	[-0.178, 0.137]	-0.26	0.79
		OppLev3	0.135	0.12	[-0.113, 0.383]	1.12	0.27
		OppLev4	0.401	0.14	[0.104, 0.698]	2.78	0.01
		EffPlaTim	0.383	0.05	[0.283, 0.483]	7.88	< .001
		BalPos	-0.147	0.03	[-0.204, -0.091]	-5.36	< .001
		TotDisOpp	0.722	0.02	[0.679, 0.765]	34.6	< .001
	LaLiga2	Constant	6.26	0.80	[4.641, 7.879]	7.82	< .001
		EffPlaTim	0.276	0.03	[0.219, 0.333]	9.79	< .001
		BalPos	-0.097	0.02	[-0.138, -0.057]	-4.85	< .001
		TotDisOpp	0.791	0.02	[0.758, 0.825]	47.8	< .001

prospective	LaLiga1	Constant	28.098	3.11	[22.006, 34.190]	9.04	< .001
		EffPlaTim	1.161	0.04	[1.076, 1.247]	26.6	< .001
		BalPos	-0.098	0.05	[-0.203, 0.007]	-1.83	0.067
	LaLiga2	Constant	29.993	1.65	[26.752, 33.233]	18.2	< .001
		EffPlaTim	1.115	0.03	[1.050, 1.180]	33.6	< .001
		BalPos	-0.054	0.03	[-0.112, 0.003]	-1.84	0.066

Note. EffPlaTim = effective playing time; BalPos = ball possession; TotDisOpp = opponent’s total distance covered during effective playing time. TeaLev = team level; OppLev = opponent level, derived from each team’s final league standing; level 1 (TeaLev1) is the reference category (not shown), so coefficients represent differences relative to level-1 teams. In LaLiga1, TeaLev1 = relegation (18^th^–20^th^, reference); TeaLev2/OppLev2 = remained (8^th^–17^th^); TeaLev3/OppLev3 = Europa League (5^th^–7^th^); TeaLev4/OppLev4 = Champions League (1^st^–4^th^). In LaLiga2, TeaLev1 = relegation (19^th^–22nd, reference); TeaLev2/OppLev2 = 13^th^–18^th^; TeaLev3/ OppLev3 = 7^th^–12^th^; TeaLev4/OppLev4 = direct promotion/play-off contenders (1^st^–6^th^).

Regarding LaLiga1, the selected model retained the team and opponent level, effective playing time, ball-possession and the opponent’s total distance, whereas MatLoc and MatOut were not selected (Equation 1). The reference category was relegation for both level variables (TeaLev1, i.e. relegation). The opponent’s distance was by far the strongest predictor (b = 0.72): each additional kilometre covered by the opponent was associated with approximately 0.72 km greater distance covered by the team. EffPlaTim added 0.38 km per min, and each minute of ball-possession subtracted 0.15 km. Champions-League-level teams ran less than relegation-level teams (-0.43 km), whereas facing a Champions-League-level opponent increased the distance covered by 0.40 km.

Equation 1:

TotDis Tea (km)=7.797+0.000×TeaLev2−0.186×TeaLev3−0.427×TeaLev4−0.020×OppLev2+0.135×OppLev3+0.401×OppLev4+0.383×EffplaTim−0.147×BalPos+0.722×TotDisOpp (1)

The re-estimated model explained a substantial proportion of the variance in total distance covered (R^2^adj = 0.8605; 10-fold cross-validated R^2^ = 0.8579), with a residual standard error of 2.386 km, a mean absolute error (MAE) of 1.860 km, and an RMSE of 2.381 km (N = 2,894). Predictions fell within 1 km of the observed value in 34.8% of matches and within 2 km in 61.9%. To test whether the previous coefficients had drifted over time, both the original model of Castellano et al. [19] and the updated model were applied to each of the five recent seasons separately (this comparison is restricted to LaLiga1, as no previous model exists for LaLiga2). The original model underestimated total distance in every season, with a positive bias ranging from +2.28 to +2.52 km (season RMSE 3.29–3.68 km), whereas the updated model showed a near-zero bias throughout (within ± 0.14 km in all seasons) and consistently lower error. When instead applied backwards to the two earliest seasons in the dataset (2019–20 and 2020–21), which precede the seasons used to fit the updated model, it overestimated total distance by 2.230 and 1.921 km per match, respectively, mirroring the underestimation the original model produces on recent seasons. Together, this season-by-season pattern shows that the discrepancy between the two models is systematic and persistent rather than confined to any single season, confirming that they reflect genuinely different levels of physical demand (Figure 1).

**FIG. 1:**
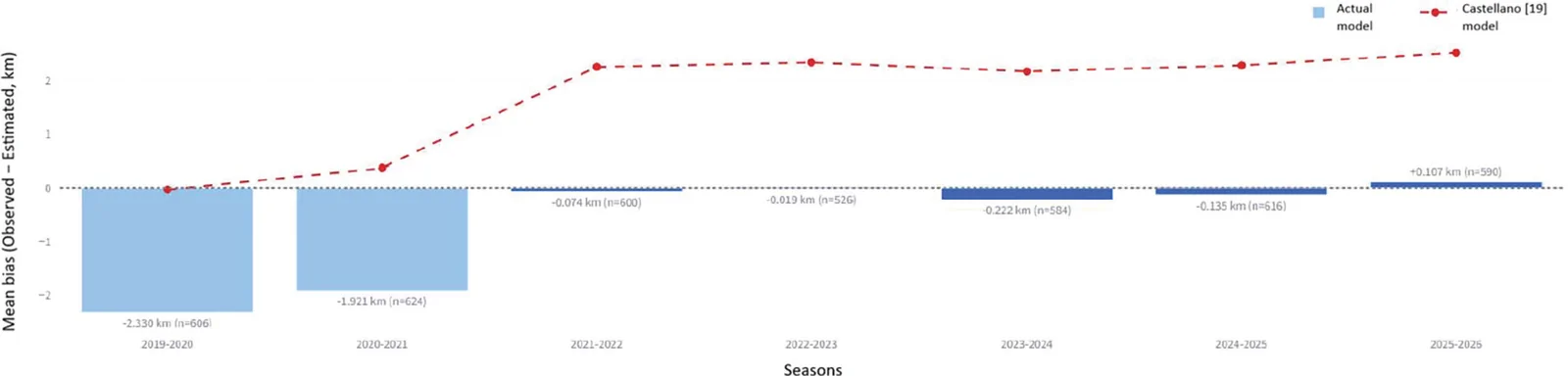
Mean prediction bias (observed–predicted total distance, km) by season for the updated model (bars) and the original model of Castellano et al. [19] (red line). Positive values indicate underestimation and negative values overestimation.

Regarding LaLiga2, the selected model was more parsimonious: only effective playing time, ball-possession and the opponent’s total distance were retained, while match location, match outcome and both level variables were not selected (Equation 2). The opponent’s distance again dominated (b = 0.79; +0.79 km per km run by the opponent), followed by effective playing time (+0.28 km per min) and ball-possession (-0.10 km per min).

Equation 2:

TotDis Tea (km)=6.260+0.276×EffPlaTim−0.097×BalPos+0.791×TotDisOpp (2)

Regarding model validity (Table 2), both models met the regression assumptions (normally distributed and homoscedastic residuals; maximum VIF = 3.69, indicating no relevant collinearity) and showed very similar explanatory and predictive capacity. For reference, the original LaLiga1 model of Castellano et al. [19] reported R^2^adj = 0.82 with the opponent’s distance and 0.64 without it; the updated models improved on both. The LaLiga2 model yielded a negative Mallows’ Cp (-3.66), indicating a highly parsimonious model whose fit was essentially equivalent to that of the full model with far fewer parameters.

**TABLE 2:** Goodness-of-fit statistics, predictive capacity and collinearity of the updated models estimated with and without the opponent’s total distance (TotDisOpp) in LaLiga1 and LaLiga2.

Indicator	With TotDisOpp		Without TotDisOpp	

	LaLiga1	LaLiga2	LaLiga1	LaLiga2
N (matches/teams/seasons)	2,894 (1,447/26/5)	3,542 (1,771/40/5)	2,894 (1,447/26/5)	3,542 (1,771/40/5)
Predictors retained (p+1)	10	4	3	3
Mallows’ Cp	7	−3.66	−1.85	−5.28
R^2^adj	0.861	0.855	0.709	0.612
Cross-validated R^2^ (10-fold)	0.858	0.853	0.704	0.610
RMSE / MAE (km)	2.38 / 1.86	2.18 / 1.73	3.45 / 2.72	3.57 / 2.79
Predictions ≤ 1 km / ≤ 2 km (%)	34.8 / 61.9	36.8 / 65.2	23.0 / 45.3	21.7 / 43.5
Maximum VIF	3.69	2.67	1.19	1.18

Note: Mallows’ Cp; R^2^adj, adjusted R-squared; RMSE, root mean square error; MAE, mean absolute error; VIF, variance inflation factor; p+1, number of predictors including the intercept.

From a descriptive and exploratory perspective, the two updated models differed mainly in their composition: TeaLev and OppLev were retained only in LaLiga1, whereas the LaLiga2 model was more parsimonious, keeping only the three predictors common to both divisions (EffPlaTim, BalPos, and TotDisOpp). For these three common predictors, the cluster-robust 95% confidence intervals overlapped between divisions, indicating that the magnitude of their association with TotDisTea was compatible across LaLiga1 and LaLiga2. Overall, the divisions differed more in which factors were retained by the model than in the size of the effects shared by both.

### Prospective models excluding opponent’s distance

To enable a prospective application, one that does not depend on the opponent’s in-match distance, a reduced model was additionally estimated for each division, excluding TotDisOpp and retaining only predictors that can be known or estimated before kick-off. These reduced models retained effective playing time and ball-possession:

Equation 3 (LaLiga1)

TotDis Tea (km)=28.098+1.161×EffPlaTim−0.098×BalPos (3)

Equation 4 (LaLiga2)

TotDis Tea (km)=29.993+1.115×EffPlaTim−0.054×BalPos (4)

As expected, removing the strongest predictor reduced explanatory and predictive capacity: the reduced models explained a lower proportion of the variance (LaLiga1: R^2^adj = 0.71, cross-validated R^2^ = 0.70; LaLiga2: R^2^adj = 0.61, cross-validated R^2^ = 0.61) and showed larger errors (RMSE = 3.45 and 3.57 km; MAE = 2.72 and 2.79 km, respectively) than the full models. Predictions fell within 2 km of the observed value in 45.3% of matches in LaLiga1 and 43.5% in LaLiga2. Their coefficients, standard errors and 95% confidence intervals, estimated with the same cluster-robust procedure, are reported in Table 1. In these reduced models effective playing time carried a substantially larger coefficient than in the full models, absorbing part of the variance previously explained by TotDisOpp, whereas ball-possession was no longer statistically significant (p = 0.07 in both divisions). These reduced equations are therefore less accurate but usable before a match, whereas the full models (Equations 1 and 2) remain the reference for retrospective analysis.

## DISCUSSION

The aim of this study was twofold: (1) to update the predictive model of the total distance covered by a team in the Spanish First Division originally proposed by Castellano et al. [19] with data from the last five seasons (2021–22 to 2025–26), and (2) to extend this approach to the Second Division. Both objectives were addressed from two complementary perspectives: a full model for retrospective evaluation and a reduced model, excluding opponent distance, for prospective pre-match estimation. Overall, the findings were broadly consistent with our expectations, although the between-model comparisons should be regarded as exploratory rather than as formally tested. TotDisOpp, EffPlaTim and BalPos were the predictors with the greatest weight in both categories, reproducing the core structure reported in the original model and reinforcing the idea that a team’s physical output is largely conditioned by its opponent and by how the game unfolds. At the same time, the models differed mainly in their composition: relative to the 2016–2020 LaLiga1 model, match location was no longer retained, and the two divisions differed in which predictors were selected. For the predictors shared by both divisions, the cluster-robust confidence intervals overlapped, so their effect sizes should be interpreted as compatible rather than as significantly different. This compatibility is itself informative: it suggests that the mechanisms linking effective playing time, ball-possession and the opponent’s running to a team’s physical output operate similarly across competitive levels, and that what distinguishes the two divisions is not the strength of these shared associations but which additional factors, competitive level in particular, carry predictive weight in each. The divergence between divisions is therefore best understood as one of model structure rather than of statistically demonstrated differences in coefficient magnitude.

The principal finding is that, in both professional divisions, the distance covered by the opponent was the dominant predictor of a team’s own total distance (b = 0.72 in the First Division and 0.79 in the Second). This reinforces the notion that football is an interactive sport in which a team’s physical output is largely conditioned by what the opponent does [19]. The stability of this coefficient over time, remaining close to the 0.71 reported for 2016–2020, suggests that the inter-team conditional relationship is an almost structural feature of the game [25], even as its absolute demands evolve. From this perspective, players’ physical performance cannot be fully understood as an isolated expression of individual or team capacities, but rather as an emergent consequence of the continuous interaction between two opposing systems. These findings highlight the need for future research and performance analysis models to explicitly account for opponent-related variables, as neglecting the interactive nature of football may lead to an incomplete understanding of the determinants of physical performance in competition.

When the temporal comparison was restricted to LaLiga1, the updated model not only improved its explanatory capacity relative to the previous equation (R^2^adj = 0.86 vs. 0.82), but also revealed changes in the relative contribution of its predictors. When coefficient magnitudes were compared, opponent distance remained the predictor with the greatest weight in the model, confirming that a team’s physical output is primarily conditioned by the locomotor behaviour of its opponent. This finding further reinforces the need to conceptualise football as an interactive system in which performance emerges from the continuous co-adaptation of two competing teams. At the same time, match location, which previously added approximately 0.28 km when playing at home, no longer contributed independently once the opponent’s running performance was considered, suggesting that part of the home advantage effect may have been indirectly capturing interactive match dynamics.

Effective playing time is one of the variables attracting the most attention recently [8, 10, 26], due to its impact on the physical performance of teams and players in competition. Among the retained predictors, effective playing time showed a slight reduction in its coefficient, from 0.42 to 0.38 km · min^−1^. This change occurs in a context of a marginal increase in effective playing time across seasons, suggesting a small expansion in the total amount of ball-in-play time during competition [8]. One possible, though speculative and untested, explanation is a dilution effect: as the baseline effective duration of matches increases, each additional minute represents a smaller proportion of the total, which could reduce its relative contribution within the model rather than reflecting a genuine loss of importance.

This mechanism, however, was not directly tested, and several alternative explanations should be considered. First, the reduced weight of effective playing time may reflect changes in tactical behaviour, if teams now manage ball-in-play time differently, weakening its direct link with total distance [10]. Second, it may stem from changes in match structure, such as stricter timekeeping and added-time computation in recent seasons, which alter the effective duration and its relationship with running output [11]. Third, methodological factors related to the tracking procedures (system calibration or data-processing changes across periods) cannot be ruled out, as has been the case in other leagues [4] Finally, part of the effect may arise from collinearity with other predictors; although the variance inflation factors in our models were moderate (maximum 3.69), some shared variance with predictors such as TotDisOpp or BalPos may partly account for the reduced coefficient. Consistent with this, effective playing time carried a substantially larger coefficient in the reduced models that excluded TotDisOpp (Table 2), where its variance inflation factor dropped to approximately 1.2. These explanations are not mutually exclusive, and disentangling them would require dedicated analyses beyond the scope of this study. In any case, effective playing time remains an important determinant of total distance covered, even if its explanatory weight has slightly decreased in favour of other contextual variables.

The third most significant contextual variable, the ball-possession of the team, exhibited the largest reduction in relative influence, decreasing from -0.27 to -0.15 km · min^−1^. This finding suggests that possession time now explains a smaller proportion of the variance in total distance covered than during the 2016–2020 period. Rather than indicating a reduction in the physical demands associated with possession, this change may reflect that possession, on its own, has become a less discriminating variable, although we advance this as a hypothesis rather than a tested result. One possible explanation is that contemporary playing styles encompass increasingly heterogeneous ways of using the ball, ranging from more direct and vertical actions to more elaborate, passing-based approaches [15, 27], so that overall possession may no longer capture these tactical differences as clearly as it once did. As teams seek to progress the ball more quickly and exploit transitions more frequently, offensive phases might involve greater locomotor demands than in previous seasons. A further hypothesis worth investigating is therefore that the physical requirements of teams in and out of possession may be converging, reducing the extent to which possession time alone differentiates total distance covered. Importantly, neither of these mechanisms was directly tested in the present study; confirming them would require analysing physical demands separately across possession phases, which lies beyond our scope and warrants dedicated investigation.

When the updated models were compared between divisions, both shared the same core predictors, effective playing time, ball-possession, and especially the opponent’s distance covered, but differed in their overall predictor structure. In both categories, the opponent’s distance was the dominant explanatory variable. Numerically, the coefficient associated with the opponent’s distance was slightly higher in LaLiga2 than in LaLiga1 (0.79 vs. 0.72); however, this difference should be interpreted as exploratory, since the cluster-robust 95% confidence intervals of the two estimates overlap and no formal test was conducted to compare them. It cannot therefore be taken as evidence of a stronger interdependence between opponents’ physical outputs in the second tier; rather, the similar magnitude in both divisions suggests that the opponent’s running is an equally influential determinant of a team’s physical output across competitive levels.

Independently of this coefficient, LaLiga2 is generally regarded as a more competitive and homogeneous environment: lower professional divisions tend to exhibit greater competitive balance and stronger contextual influence on performance [28]. This higher parity, together with the more compressed range of team levels in LaLiga2 and the narrower margins that decide promotion and relegation, which make final league position a weaker proxy for physical performance [29], may help explain why team and opponent level were not retained as predictors in this division, yielding a more parsimonious model than in LaLiga1.

With regard to the second most significant variable in the predictive equation, EffPlaTim, its weight was somewhat lower in LaLiga2; the mechanisms underlying this difference are not yet fully understood and are likely multifactorial, particularly given that effective playing time values are lower in this division [8]. Only the First Division model retained indicators of competitive level, for both the team and its opponent. In the LaLiga1 model, higher-level teams were associated with a lower total distance than their lower-level counterparts. One possible interpretation is that stronger teams meet the competitive demands of a match with a comparatively lower locomotor response, in line with previous research reporting that higher-ranked teams accumulate more passes and sustain higher ball-possession [15]. This interpretation should, however, be treated with caution: it is not directly demonstrated by our regression, the team-level coefficients were not individually significant under cluster-robust inference, and the association may partly reflect residual confounding by possession style, match state, tactical strategy or team identity.

Several limitations should be acknowledged. The level variable was not strictly homologous between divisions (European places in the First Division versus promotion/play-off positions in the Second), so between-league comparisons of this predictor must be made cautiously; in any case, it did not enter the Second Division model. Furthermore, competitive level, being based on final league standings, is a retrospective, season-level descriptor rather than information available before each match; a genuine pre-match use of the full LaLiga1 model would therefore require this level to be approximated (e.g., from the standings at that point in the season or from pre-season expectations). This constraint does not apply to the reduced prospective models, which rely only on effective playing time and ball-possession. The models estimate the team’s aggregate total distance and do not capture how that demand is distributed across individual players or playing positions. Because matches involving a sending-off were excluded from model estimation, the equations apply only to matches played under regular 11-versus-11 conditions and should not be used to evaluate or anticipate physical demands in games where a player has been dismissed. Finally, the present model did not account for other situational variables that fluctuate within a match (e.g., numerical superiority or inferiority after red cards, or substitutions), nor for broader contextual variables that frame each fixture (e.g., opponent’s recent form, fixture congestion, travel demands, match importance, or environmental conditions). Incorporating these dynamic and context-dependent factors in future studies would allow a more precise modelling of physical performance and a better understanding of the conditions under which competitive level exerts its effect.

## CONCLUSIONS

Updating and extending the model of Castellano et al. [19] confirms that, across both Spanish professional divisions, a team’s total distance and that of its opponent were strongly associated within matches, underscoring the interactive nature of football. The two categories differ, however, in their predictive structure: competitive level contributes in the First Division but is dispensable in the Second, where a parsimonious model based on effective playing time, ball-possession and the opponent’s distance suffices. The re-estimation also shows that the original coefficients had drifted, match location was no longer retained and the weight of ball-possession decreased, which justifies periodically refreshing this type of model rather than assuming it is time-invariant.

The study provides two complementary tools, valid provided their use is accompanied by robust grouped and temporal validation such as that applied here: the full equations, which include the opponent’s distance, and the reduced equations, which exclude it. Their applied scope depends on when they are used. After a match, the opponent’s observed distance is entered directly into the full equation, providing the most accurate benchmark against which a team’s physical output can be evaluated. Before a match, when the opponent’s actual distance is not yet available, two options are possible: its value can be replaced by an expected estimate, derived from recent form, historical records or the opponent’s competitive profile, and entered into the full equation, preserving its higher accuracy but inheriting the uncertainty of that estimate; or the reduced equations can be applied, which remove the need to estimate the opponent’s distance and are therefore simpler, although less precise. It should be noted, however, that the reduced equations are not entirely free of prior estimation: because the observed values of effective playing time and ball-possession are only available during or after the match, their prospective application still requires expected pre-match values for these two predictors. The choice therefore depends on whether a reliable pre-match estimate of the opponent’s running is available and on the accuracy required. Consequently, the accuracy reported here, obtained using observed values of effective playing time and ball-possession, represents a best-case estimate: in a fully prospective setting these inputs must themselves be estimated, and the resulting uncertainty would tend to reduce predictive accuracy, with individual predictions departing from observed values in either direction. Ultimately, this residual uncertainty is not merely a statistical artefact but a reflection of the game itself: the physical demands of a match are not fixed in advance, but are written as the match is played.
